# At the Crossroad Between Resiliency and Fragility: A Neurodevelopmental Perspective on Early-Life Experiences

**DOI:** 10.3389/fncel.2022.863866

**Published:** 2022-04-07

**Authors:** Gabriele Chelini, Luca Pangrazzi, Yuri Bozzi

**Affiliations:** ^1^CIMeC-Center for Mind/Brain Sciences, University of Trento, Rovereto, Italy; ^2^Consiglio Nazionale delle Ricerche (CNR) Neuroscience Institute, Pisa, Italy

**Keywords:** early-life stress, adverse childhood experience, environmental enrichment, neurodevelopment, critical periods, psychiatric disorders, inflammation

## Abstract

Postnatal development of the brain is characterized by sensitive windows during which, local circuitry are drastically reshaped by life experiences. These critical periods (CPs) occur at different time points for different brain functions, presenting redundant physiological changes in the underlying brain regions. Although circuits malleability during CPs provides a valuable window of opportunity for adaptive fine-tuning to the living environment, this aspect of neurodevelopment also represents a phase of increased vulnerability for the development of a variety of disorders. Consistently, accumulating epidemiological studies point to adverse childhood experience as a major risk factor for many medical conditions, especially stress- and anxiety-related conditions. Thanks to creative approaches to manipulate rodents’ rearing environment, neurobiologist have uncovered a pivotal interaction between CPs and early-life experiences, offering an interesting landscape to improve our understanding of brain disorders. In this short review, we discuss how early-life experience impacts cellular and molecular players involved in CPs of development, translating into long-lasting behavioral consequences in rodents. Bringing together findings from multiple laboratories, we delineate a unifying theory in which systemic factors dynamically target the maturation of brain functions based on adaptive needs, shifting the balance between resilience and vulnerability in response to the quality of the rearing environment.

## Introduction

Early-life experiences gained a lot of attention in the context of psychosocial development since the early theories formulated by the founding fathers of developmental psychology (namely, Piaget, Vygotsky, and Bowlby) (Baltes et al., 1980; McLeod, 2017). According to them, the quality of the rearing environment is a critical component for individuals’ psychological development, which significantly contributes to reaching a good quality of adult life. This central pillar of 20th-century psychology was recently corroborated by epidemiological studies, which identified adverse childhood experiences (ACEs) as a major risk factor for a wide range of severe medical conditions such as psychiatric disorders, cancer, risk-taking behaviors, and more (Center for disease control and prevention [CDC], 1998; Felitti et al., 1998). Consistently, a large body of evidence identified time-window where postnatal brain development is exceptionally sensible to environmental factors; namely, the critical periods (CPs) (Reh et al., 2020). During these windows, local circuitry is characterized by an enhanced form of synaptic plasticity (juvenile plasticity), reshaping the brain in an experience-dependent fashion (Hensch, 2004; Reh et al., 2020). Multiple relevant physiological processes occur during CPs, such as changes in synaptic number and morphology (Holtmaat et al., 2005; Holtmaat and Svoboda, 2009; Wen and Barth, 2011), myelination (Fletcher et al., 2021), and maturation of local inhibitory circuitry (Hensch, 2005; Takesian and Hensch, 2013). To this last point, a special role is fulfilled by inhibitory interneurons expressing the calcium binding protein parvalbumin (PV-cells) (Hensch, 2005; Takesian and Hensch, 2013). Physiological maturation of PV-cells occurs drastically during CPs and completes coincidentally with the closure of juvenile plasticity (Hensch, 2005; Takesian and Hensch, 2013; Reh et al., 2020). A critical milestone of PV-cells maturation, during CPs, is the emergence of specialized extracellular matrix structures known as perineuronal nets (PNNs) (Pizzorusso et al., 2002; Berardi et al., 2004). In adult rodents, enzymatic removal of PNNs reverts PV-cells physiology to an immature state, re-instating a juvenile-like form of plasticity (Pizzorusso et al., 2002; Hensch, 2004; Wingert and Sorg, 2021). Although providing an intrinsic ontogenetic advantage (improving adaptation to the environment, hence survival chances), CPs also represent phases of increased vulnerability to environmental insults. Understanding the impact of early life experiences on sensitive periods of development is a primary challenge in neurobiology because it provides fundamental clues for the prevention and intervention of brain disorders. To do so, neuroscientists developed creative paradigms–for manipulating the quality of early-life in laboratory animals, either positively or negatively.

In this article, we briefly summarize the state of the art of research on the neurobiology of early-life experience in rodents, focusing on the role of CPs of development and their unique location at the intersection between resiliency and vulnerability for brain disorders.

### Multiple Approaches to Study Early-Life Experiences

**FIGURE 1:**
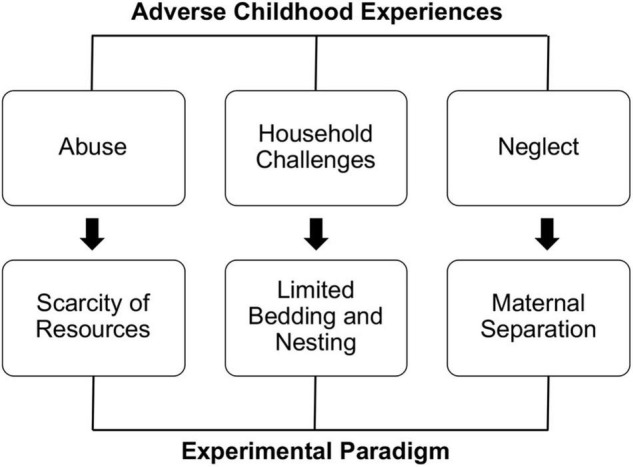
Different kinds of ACEs require elective experimental approaches.

In the field of early-life experience, the advantage of studying laboratory animals is that it allows you to compare alternative rearing conditions with respect to the standard conditions commonly used in animal facilities. To do so, neuroscientists developed standardized approaches to mimic positive or negative environmental conditions affecting early-life development. Specifically, the impact of the positive experience was mostly studied using environmental enrichment (EE) paradigms (Cancedda et al., 2004). In opposition with EE, with the idea of exploring the consequences of the lack of a stimulating environment, some laboratories have investigated the effect of environmental impoverishment (EI) (Narducci et al., 2018). Finally, the study of early-life adversities (eLA) required several alternative approaches, as the quality of ACEs can present in a spectrum of different insults (Center for disease control and prevention [CDC], 1998; Felitti et al., 1998; Brenhouse and Bath, 2019; Figure 1). A common feature of all these approaches is the critical mediation of maternal care. Dams are single-handedly responsible for the pups’ survival during the first 2 weeks of offspring’s life, providing nutrition, hygiene care, and preventing hypothermia. Manipulation of rearing conditions drastically impact the mothers’ neurobiology and behavior, disrupting the quality of maternal care and resulting in long-lasting neurobiological and behavioral consequences for the offspring’s life (Guzzetta et al., 2009; Sale et al., 2014; Luchetti et al., 2015; Couto-Pereira et al., 2016, 2019; Walker et al., 2017; Narducci et al., 2018; Brenhouse and Bath, 2019; Gallo et al., 2019; Sparling et al., 2020).

#### Early Environmental Enrichment and Impoverishment

The early environmental enrichment (eEE) paradigm consists of large cages containing several animals to foster social interactions, objects of various shapes and sizes to stimulate curiosity, and running wheels or similar tools to promote physical activities (Cancedda et al., 2004; Baroncelli et al., 2010). These features of eEE cages can be applied simultaneously, or one at a time, depending on the experimental questions the investigator aims to ask. To understand how maternal behavior might positively promote neurodevelopment in the offspring, a brilliant study in 2009 showed that gentle tactile stimulation (simulating maternal grooming) mimics the effect of eEE, resulting in overlapping beneficial effects on newborn rats (Guzzetta et al., 2009). Opposite to the eEE, early environmental impoverishment (eEI) consists of small cages with opaque walls to impoverish visual stimulation, lack of interactive objects and tools for physical activity (Narducci et al., 2018). In most cases, exposure to eEE and eEI begins on the pups’ birthday (Cancedda et al., 2004; Baroncelli et al., 2010; Narducci et al., 2018) and can last for a variable amount of time, depending on the specificity of the experimental question. In some cases, eEE was used later during preweaning development (Kohl et al., 2002; Hegde et al., 2020).

#### Early-Life Adversities

According to the most extensive epidemiological study on childhood adversities, ACEs can present within three umbrella macro-categories: household challenges, abuse, and neglect (Felitti et al., 1998). These conditions can be operationally applied to laboratory mice using specific approaches: limited bedding and nesting (household challenges), scarcity of resources (abuse), and maternal separation (neglect) (Brenhouse and Bath, 2019; Figure 1).

In the “limited bedding and nesting” (LBN) approach, dams and pups are transferred to a cage with restricted availability of nesting material. In addition, direct contact with the bedding is prevented by installing an aluminum grid about 2.5 cm from the bottom of the cage. This approach has been shown to fragment maternal care, inducing hypervigilance and hyperactivity in mothers (Walker et al., 2017; Brenhouse and Bath, 2019).

Similar to LBN, the “scarcity of resources” (SR) paradigm partially removes the nesting material from the home cages. Studies with this approach report increased aggressiveness and abusive-like behaviors of dams against the pups (Brenhouse and Bath, 2019; Keller et al., 2019). Although more empirical evidence is required to determine the entity of maltreatments and validity of this approach, it appears to be a potential ecological and non-invasive tool for modeling parental abuse in rodents. Given the technical similarity of LBN and SR, it is surprising to see such a divergent impact on maternal care. One possible interpretation could be that on the one hand LBN dams are forced to re-adapt to a virtually novel environment, triggered by a massive change of the living space. On the other hand, SR does not call for a generalized re-adaptation process. Given this interpretation, LBN selectively leads to anxious hypervigilance given by the unpredicted novelty; on the contrary, nest scarcity would be most associated with the frustration of the loss, leading to increased anger and aggression.

Maternal separation (MS) is vastly used and validated and consists in the removal of the dam from the pup’s cage for extended periods (at least 3 h) (Brenhouse and Bath, 2019; Rocha et al., 2021). While very easy to apply, this approach must be used with meticulous attention, as confounding factors like malnutrition, drop in body temperature, and poor hygiene can dramatically influence experimental outcomes, resulting in contrasting findings across different laboratories (Brenhouse and Bath, 2019).

It is essential to specify that all the mentioned stressful events can be applied for short (1–3 days) or long periods (up to 1 week) as well as intermittently and at different stages of early development (between P1 and P15, virtually corresponding to the first trimester of human postnatal age). The duration and frequency of stressors as well as the age of the pup at the time of exposure, have a critical impact on the long-lasting behavioral and neurobiological consequences, explaining some discrepancies observed within the same paradigms (Brenhouse and Bath, 2019). Lastly, a critical aspect that emerged in eLA studies consists in significant sexually dimorphic phenotypes (Couto-Pereira et al., 2019; Bath, 2020; Faraji et al., 2022). These findings are of particular relevance, given the well-established sex-bias observed in epidemiological studies on brain disorders (Altemus et al., 2014; Bangasser and Valentino, 2014; Kokras and Dalla, 2014; Zuena et al., 2016) and support the ecological validity of eLA approaches in rodents.

### Early-Life Experience Shapes the Brain During Critical Periods of Development

Following early theories of psychosocial development (Baltes et al., 1980), animal studies confirmed the central dogma where positive caregiving and stimulating early-life predisposes to a healthier life. Specifically, early-life experiences were shown to impact postnatal CPs drastically. Not surprisingly, bidirectional impact on CPs occurs in animals exposed to eEE, eEI, or eLA. For instance, exposure to an eEE promotes the maturation of primary sensory systems (visual, somatosensory, and auditory) from a molecular and functional point of view. In some cases, this maturation is accompanied by enhanced perceptual capacity compared to standard-reared controls (Coq and Xerri, 2001; Cancedda et al., 2004; Cai et al., 2009; Xu et al., 2009; Baroncelli et al., 2010; Zennou-Azogui et al., 2016). Better cognitive performances (Baroncelli et al., 2010; Sale et al., 2014) and reduced anxiety (Baldini et al., 2013; Hegde and Mitra, 2020) generally accompany these effects, providing beneficial consequences in the rodent’s model of neurodevelopmental disorders (Lonetti et al., 2010; Chakrabarti et al., 2011; Sale et al., 2014; Yamaguchi et al., 2017; Consorti et al., 2019) and cognitive decline (Petrosini et al., 2009). Cortical development is consistently delayed in animals reared in eEI, coherently with impaired cognitive performances (Narducci et al., 2018).

Given these findings, the protracted consequences of eLA on adulthood emotional regulation are less intuitive. On one hand, increased anxiogenic behaviors and altered fear regulation are consistently detected in adult rodents with history of eLA, irrespectively from the kind of stressors (Brenhouse and Bath, 2019; Guadagno et al., 2020; Manzano Nieves et al., 2020; Tsotsokou et al., 2021). On the other hand, surprising findings are observed in the cortico-limbic pathway. For instance, an increased density of PV interneurons in the basolateral amygdala was reported in young mice reared with LBN (Manzano Nieves et al., 2020). Similarly, MS resulted in increased density of PNNs enveloping PV-cells in adult male mice prefrontal cortex (Gildawie et al., 2020). Consistently, another study found hypertrophic dendritic branching in adult mice exposed to MS, an abnormality that was successfully rescued by pre-weaning EE (Koe et al., 2016). Notably, optogenetic studies showed that correcting PV-function in LBN animals was able to re-instate conditioned fear behavior analogous to standard reared mice (Manzano Nieves et al., 2020).

These findings show that postnatal stress directly impacts some of the key factors contributing to CPs of development (namely, PV-cells and PNNs), qualifying them as primary candidates to mediate long-lasting behavioral abnormalities. Moreover, most of these studies show a gain-of-function in both PV and PNNs, suggesting that eLA might trigger an early and empowered maturation of limbic regions, analogous to what is seen in sensory regions in response to eEE. In light of these evidence, we postulate that early-life experiences might not induce, *per se*, substantial changes in the levels of specific cellular factors, but rather bias the allocation of resources in favor of targeted systems under the direct guidance of external cues.

Our hypothesis calls for some underlying mediators to control resource allocation across different brain regions in an experience-dependent manner. A primary candidate for this role is the neuroendocrine system. The endocrine glands secrete systemic hormones in response to stress (corticosterone) or pleasure (oxytocin) and activate selected cellular programs upon targeted cellular signaling. Transversal evidence corroborates this hypothesis: levels of oxytocin are found to increase in female mice reared under eEE conditions (Cutuli et al., 2019; Faraji et al., 2022), while intranasal administration of oxytocin reverses the impairment in social behavior consequent to MS (Joushi et al., 2021). On the contrary, enriched early-life experience significantly reduces the expression of corticosterone in peripheral blood coherently with accelerated cortical development (Guzzetta et al., 2009). Moreover, a higher level of corticosterone is observed in pups exposed to LBN, MS, and SR (Heun-Johnson and Levitt, 2016; Walker et al., 2017; Joushi et al., 2021).

The role of neurotrophins and growth factors is less clear. While molecules such as brain-derived neurotrophic factor (BDNF) and insulin-like growth factor 1 (IGF-1) were established as positive modulators of eEE in sensory systems’ development (Cancedda et al., 2004; Guzzetta et al., 2009; Baroncelli et al., 2010), their role in response to eLA is still unclear. According to the findings of accelerated maturation of limbic regions as a consequence of LBN and MS, it is reasonable to hypothesize a targeted increase of BDNF and IGF-1 in the cortico-limbic pathway. Two separate studies on MS-rats corroborate this hypothesis: one showing widespread cortical elevation of both BDNF and IGF-1 (Lee et al., 2012); another reporting elevated BDNF levels in the prefrontal cortex (Poleksic et al., 2021). In the amygdala, however, no significant changes in BDNF expression were observed (Hegde et al., 2020). Given the controversy, further investigation is required to determine the specific contribution of growth and neurotrophic factors to the development of cortico-limbic pathway in eLA models (Studies showing early-life experience impact on CPs are summarized in Supplementary Table 1).

Another additional aspect, potentially contributing to the consequence of eLA onto developmental process, is the involvement of multi-systemic inflammation and oxidative stress, two interconnected factors that have gained central attention in neuropsychiatric research over the past decade (Schafer and Stevens, 2010; Sekar et al., 2016; Devlin et al., 2021; Dziabis and Bilbo, 2021). On the one hand, the involvement of the immune system has been extensively described in a variety of brain disorders (Aw et al., 2021). On top of that, the intimate and bidirectional relationship between neuroinflammation and oxidative stress was shown to have a mechanistic implication on PV-cells and PNNs (Cabungcal et al., 2013; Soares et al., 2020). Indeed, elevated levels of pro-inflammatory molecules were extensively described in people with a history of ACE (Dutcher et al., 2020). For instance, a very elegant study described an association between ACEs and impaired pro-inflammatory glucocorticoid signaling (Danese et al., 2007). Thanks to this study, it is estimated that around 10% of cases of low-grade inflammation within the population, measured with C-reactive protein (CRP) levels in the blood, could be attributed to childhood maltreatment. Moreover, the elevated blood level of interleukin (IL) 6 and CRP were found in people with a history of ACE, when exposed to acute stressful events (Baumeister et al., 2016). Similarly, a systematic review established that mice exposed to MS present a short-term increased in the expression of IL-6, tumor necrosis factor-alpha (TNF), and IL-10 in non-blood tissues. At the same time, the levels of these molecules did not change in the blood (Dutcher et al., 2020). However, when further stress was applied, MS animals showed significant overexpression of IL-6, IL-1β, TNF, and IL-10 in the blood, closely matching previous findings in humans (Dimatelis et al., 2012). Despite the limited viability of studies in this area, a similar tendency of increased neuro-inflammation was observed in LBN animals; a clear example of this shows altered immune responses to amyloid plaques in a mouse model Alzheimer’s disease previously exposed to LBN (Hoeijmakers et al., 2015, 2017; Lesuis et al., 2016). On the other hand, eEE has extensively been described as protective against neuroinflammation (Jurgens and Johnson, 2012; Muscat and Barrientos, 2020; Reddaway and Brydges, 2020). Hence, converging findings from the human population and animal models suggest that eLA contributes to the onset of chronic inflammatory processes, representing an instrumental vulnerability factor leading to the development of a broad spectrum of neuropathological conditions in adulthood.

We would like to emphasize that, as nefarious as inflammation is for neurodevelopment, it also offers valuable therapeutic landscapes. Attenuating inflammatory processes might be a beneficial clinical option during early developmental stages, in conjunction with non-pharmacological approaches such as cognitive-behavioral therapies. This concept was empirically confirmed by a recent work showing early administration of *N*-acetylcysteine in conjunction with eEE significantly reduces neuroinflammation, rescuing abnormalities of GABAergic function and PNNs in a mouse model with an early oxidative insult (Dwir et al., 2021).

**FIGURE 2:**
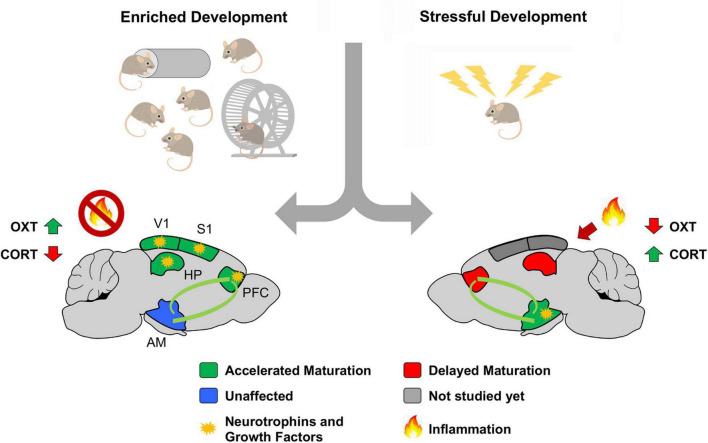
Theoretical model summarizing the impact of early life experiences on postnatal brain development. We propose a model where early-life experience biases the allocation of systemic factors (putatively neurotrophins and growth factors) based on adaptive needs. Hypertrophy of regions contributing to emotional regulation might result in maladaptive states of hypervigilance, stress and anxiety, laying the foundation for a variety of psychiatric conditions. Abbreviations: AM, Amygdala; CORT, corticosterone; HPP, hippocampus; OXT, oxytocin; PFC, prefrontal cortex; S1, primary somatosensory cortex; V1, primary visual cortex. Adapted from https://it.dreamstime.com/.

Results here described are graphically summarized in Figure 2.

## Discussion

Reporting that the maturation of limbic regions is positively modulated by eLA might be interpreted as a controversial finding, contrasting with epidemiological studies on mental illness. We want to extrapolate a theoretical interpretation of this gap based on our current knowledge. Intuitively, a brain development biased in emotional response has a decisive survival advantage in the wild. More robust and faster sympathetic activation, triggered by a hypervigilant limbic system, provides the subject an enhanced perception of danger and hasty behavioral response. This condition might not be ideal in modern human society, where mere survival instincts are, in most cases, neither a necessity nor a priority. In this context, improved threat perception might translate into detrimental consequences for everyday life, leading to unmanageable anxiety, uncontrollable mood, and maladaptive behaviors. A partial confirmation of this theory comes from studies showing that eEE, in addition to the benefits mentioned above, results in a pronounced degree of individuality among littermates’ mice (Zocher et al., 2020). This data suggests that a stimulating environment contributes to the behavioral diversification of subjects with similar genetic backgrounds, thus supporting a context-based behavioral adaptation rather than threat-assessment decision-making. Accordingly, a recent meta-analysis showed how EE has a potent impact in reversing the negative consequences of stress onto cognitive performances, highlighting the intrinsic capacity of the brain to shift between resiliency and vulnerability under the driving force of experience (Macartney et al., 2022).

According to our hypothesis, it is worth highlighting the fact that both resiliency and vulnerability can be relative concepts when looking through the lens of an evolutionary-adaptive perspective. For instance, a study showed how stress and anxiety can be found increased in EE animals, putatively providing better risk-assessment behavior (Dos Anjos-Garcia et al., 2022). As such, hypothetically, eLA might result in adaptive responses under certain stressful circumstances. In the future, more naturalistic approaches might be employed to better investigate this dynamic balance of adaptive behaviors.

In conclusion, current literature on early-life experience is well incorporated in the modern clinical conceptualization of mental illnesses as described in the Diagnostic and Statistical Manual of Mental Disorders (DMS-5; American Psychiatric Association, 2013).

Lastly, we care to bring one final point to the scientific community’s attention aiming to establish reliable preclinical models of mental illness. As the study of early-life experience in rodents has been used to understand the environmental contribution to human mental disorders, it is essential to remind ourselves of the fundamental role of genetic vulnerability in the etiology of virtually every psychiatric condition (Sullivan and Geschwind, 2019). Thanks to the critical advancement of genetic engineering, it is now possible to generate rodents’ models with different degrees of genetic vulnerability (full knock-out, heterozygous mutations, or partial genetic depletion). These models can be combined with all the mentioned approaches to study early-life experiences. Investigating the interplay between genetic background and early-life experience offers the opportunity to explore how environmental factors shift the balance between resilience and vulnerability in the context of psychological wellbeing, providing novel critical insights in the pathophysiology of mental illnesses.

## Permission to Reuse and Copyright

Some of the graphical contents were legally and freely acquired from https://it.dreamstime.com/ and re-adapted to the figures.

## Author Contributions

GC conceptualized and wrote the manuscript. LP wrote the paragraph on inflammation. All authors edited the manuscript, contributed to the article, and approved the submitted version.

## Conflict of Interest

The authors declare that the research was conducted in the absence of any commercial or financial relationships that could be construed as a potential conflict of interest.

## Publisher’s Note

All claims expressed in this article are solely those of the authors and do not necessarily represent those of their affiliated organizations, or those of the publisher, the editors and the reviewers. Any product that may be evaluated in this article, or claim that may be made by its manufacturer, is not guaranteed or endorsed by the publisher.
