# Supplementary material for: At the Crossroad Between Resiliency and Fragility: A Neurodevelopmental Perspective on Early-Life Experiences
Source: Front Cell Neurosci. 2022 Apr 7;16:863866. doi: 10.3389/fncel.2022.863866 (PMC9023311; doi:10.3389/fncel.2022.863866)
Supplement: Supplementary file 1 [file Data_Sheet_1.PDF]

		Consequences		
**Approach**	**Species**	**Cellular/Molecular**	**Functional/Behavioral**	**Reference (PMID)**
Early EE	Mouse	↖ BDNF/Gad65-67 – V1	↖ VEP	15152044
Massages (tactile enrichment)	Rat<br>Human	↖ Igf1 – V1<br>↓ CORT - Blood	↖ VEP	19420271
Pre-weaning EE (post-MS)	Rat	↓ CORT - Blood<br>↓ GR/BDNF - Amy<br>≀ Spine Density - Amy	≀ Anxiety	32820184
Early EI	Rat	↙ Igf1 – GAD67 – V1<br>↙ Myelination – V1	↙ VEP<br>↓ Cognitive Functions	20172507
LBN	Mouse	↑ PNNs - Amy<br>↓ GluN1/GluA2 - Amy	↑ PV-Cells activity<br>↑ Fear<br>↑ LTP - Am	32978287
LBN	Mouse	↖ Pv-Cells- Amy	↙ Fear Expression<br>↑ Anxiety	32692310
MS (brief)	Rat	↑ mGluR5 - mPFC	↓ Anxiety	34137089
MS (long)	Rat	-	↑ Anxiety	34137089
MS	Rat	↓ PV-Cells/PNNs – iPFC<br>↑ PV-Cells/PNNs – pPFC<br>↑ PNNs - Amy	-	31887358
MS	Rat	↑ CORT - Blood<br>↑ GR/BDNF - Amy<br>↑ Spine Density - Amy	↑ Anxiety	32820184

↖ Accelerated; ↑ Increased (Adult); ↙ Delayed; ↓ Decreased (Adult); ≀ Rescued

Supplementary Table 1.
Summary of studies showing the effect of early-life experience onto neurobiological correlates of CPs, resulting in long-lasting functional and behavioral consequences.
List of abbreviations not previously stated:
VEP = Visual evoked potentials. **LTP** = Long Term Potentiation. **m**=medial / **i**=infralimbic / **p**=prelimbic **PFC. Amy=** Amygdala**. GR=** Glucocorticoid Receptor. **CORT=** Corticosterone. **GluN1=** ionotropic glutamate receptor-N1. **GluA1=** ionotropic glutamate receptor-A1. **GAD**= glutamic acid decarboxilase.
